# Revisiting the role of Substance P and CGRPα

**DOI:** 10.7554/eLife.106766

**Published:** 2025-04-16

**Authors:** Weihua Cai, Arkady Khoutorsky

**Affiliations:** 1 Department of Anesthesia and Faculty of Dental Medicine and Oral Health Sciences, McGill University Montreal Canada

**Keywords:** pain, somatosensation, neuropeptides, Mouse

## Abstract

Mice lacking two neuropeptides thought to be essential for processing pain show no change in how they respond to a wide range of harmful stimuli.

**Related research article** MacDonald DI, Jayabalan M, Seaman JT, Balaji R, Nickolls AR, Chesler AT. 2025. Pain persists in mice lacking both Substance P and CGRPα signaling. *eLife* **13**:RP93754. doi: 10.7554/eLife.93754.

Feeling pain is crucial to survival. It helps us avoid dangerous situations and direct our attention to injured body parts, preventing further damage and promoting healing. Given the importance of pain sensation, diverse and often redundant mechanisms have evolved to ensure the endurance of pain transmission. This built-in compensatory system, however, also makes pathological pain particularly difficult to treat.

For decades, scientists have believed that neuropeptides (a type of messaging compounds released by neurons) play an important role in pain. Substance P and calcitonin gene-related peptide (CGRPα), in particular, are both highly expressed in sensory neurons that transmit pain signals from the periphery of the body to the spinal cord. Their release contributes to inflammation at the site of an injury by activating immune and endothelial cells. At the spinal cord, they enhance pain transmission by increasing the activity of local neurons (Figure 1; Zieglgänsberger, 2019; Russo, 2017). These roles have sparked extensive research examining Substance P and CGRPα as potential targets for pain relief. Yet, targeting the expression of these two molecules in animal models of somatic and visceral pain has led to variable and often inconsistent results (Cao et al., 1998; Salmon et al., 2001; Woolf et al., 1998).

**Figure 1.**
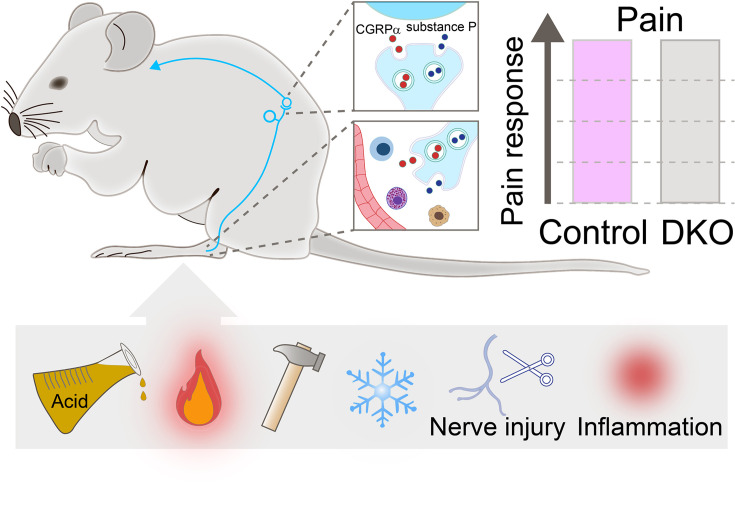
Pain responses in double knockout mice lacking genes encoding for Substance P and CGRPα. Neurons (cyan string-like structure) that relay pain signals from the periphery of the body to spinal cord cells (cyan curved arrow) release messaging signals in the form of neuropeptides, such as Substance P (blue small circles) and CGRPα (red small circles). In the spinal cord (top inset), the neuropeptides increase pain signals; at the periphery (bottom inset), they act on immune cells (shown in blue, purple, and yellow) and blood vessels (red structure) to activate inflammation. Double knockout (DKO) mice, in which both neuropeptides are missing, were exposed to painful stimuli (acid, heat, mechanical, and cold), received nerve injury and went through inflammation (two sources of chronic pain). Their pain responses (grey bar in chart) did not differ from those of control animals (purple bar).

Inhibiting CGRPα has shown positive effects in animal models of migraine, and several therapeutics that block CGRPα or its receptor are now approved for treating this condition (Charles and Pozo-Rosich, 2019). However, these treatments have not been useful for other pain conditions so far. Meanwhile, inhibiting the receptor for Substance P failed to relieve chronic pain in clinical trials (Hill, 2000). In response, researchers have suggested that the overlap between Substance P and CGRPα, which are found in the same pain-transmitting neurons, may explain why blocking one neuropeptide alone leads to minimal pain relief. This may especially be the case in genetic models where compensatory mechanisms may arise during development. But what if, instead, these neuropeptides are not as essential for mediating pathological pain as we thought?

Now, in eLife, Alexander Chesler and colleagues from the National Institutes of Health – including Donald Iain MacDonald as first author – report having addressed the potential redundancy between Substance P and CGRPα (MacDonald et al., 2025). The team generated a double knockout mouse lacking the genes encoding for both neuropeptides. They first confirmed the absence of Substance P and CGRPα expression using both immunostaining and ‘sniffer cells, which are genetically modified to react to the presence of a specific neuropeptide by exhibiting an increase in intracellular calcium.

MacDonald et al. then subjected both mutant and wild-type mice to behavioural tests, revealing no differences in their sensitivity to mechanical, heat, cold, chemical, and visceral pain stimuli. In addition, both groups of animals had the same response to pain caused by inflammation, peripheral nerve injury and chemotherapy treatment (Figure 1). Finally, the double knockout mice displayed similar levels of inflammation as animals that still carry Substance P and CGRPα genes. These findings suggest that these neuropeptides play a non-essential role in pain, challenging the notion that the compensatory activities of Substance P and CGRPα account for the lack of strong pain phenotypes in animals carrying the gene for only one of the two neuropeptides.

However, additional possibilities cannot be ruled out, including potential redundancy with other mechanisms that have similar functions. Moreover, Substance P and CGRPα impact the spinal cord and brain differently, increasing pain signals in the former and decreasing them in the latter (Zieglgänsberger, 2019; Paige et al., 2022; Huang et al., 2000). These two opposing effects may mask each other in mice in which both neuropeptides are absent from the entire nervous system. Functional compensation is also more likely to arise during development in knockout animal models, due to the target genes being absent from the start (Woolf et al., 1998). In this regard, genetically manipulating or functionally inhibiting the neuropeptides in specific brain areas and cell types during adulthood would help strengthen the results of this important study. Sex differences might be also at play as CGRPα in the spinal cord induces stronger pain signals in females (Paige et al., 2022). Overall, the findings of the study reignite the discussion on the robustness of the pain pathway’s organization, where multiple mechanisms ensure the persistence of pain sensation.

## References

[bib1] Cao YQ, Mantyh PW, Carlson EJ, Gillespie AM, Epstein CJ, Basbaum AI (1998). Primary afferent tachykinins are required to experience moderate to intense pain. Nature.

[bib2] Charles A, Pozo-Rosich P (2019). Targeting calcitonin gene-related peptide: a new era in migraine therapy. Lancet.

[bib3] Hill R (2000). NK1 (substance P) receptor antagonists--why are they not analgesic in humans?. Trends in Pharmacological Sciences.

[bib4] Huang Y, Brodda-Jansen G, Lundeberg T, Yu LC (2000). Anti-nociceptive effects of calcitonin gene-related peptide in nucleus raphe magnus of rats: an effect attenuated by naloxone. Brain Research.

[bib5] MacDonald DI, Jayabalan M, Seaman JT, Balaji R, Nickolls AR, Chesler AT (2025). Pain persists in mice lacking both Substance P and CGRPα signaling. eLife.

[bib6] Paige C, Plasencia-Fernandez I, Kume M, Papalampropoulou-Tsiridou M, Lorenzo L-E, David ET, He L, Mejia GL, Driskill C, Ferrini F, Feldhaus AL, Garcia-Martinez LF, Akopian AN, De Koninck Y, Dussor G, Price TJ (2022). A female-specific role for calcitonin gene-related peptide (CGRP) in rodent pain models. The Journal of Neuroscience.

[bib7] Russo AF (2017). Overview of neuropeptides: awakening the senses?. Headache.

[bib8] Salmon AM, Damaj MI, Marubio LM, Epping-Jordan MP, Merlo-Pich E, Changeux JP (2001). Altered neuroadaptation in opiate dependence and neurogenic inflammatory nociception in alpha CGRP-deficient mice. Nature Neuroscience.

[bib9] Woolf CJ, Mannion RJ, Neumann S (1998). Null mutations lacking substance: elucidating pain mechanisms by genetic pharmacology. Neuron.

[bib10] Zieglgänsberger W (2019). Substance P and pain chronicity. Cell and Tissue Research.

